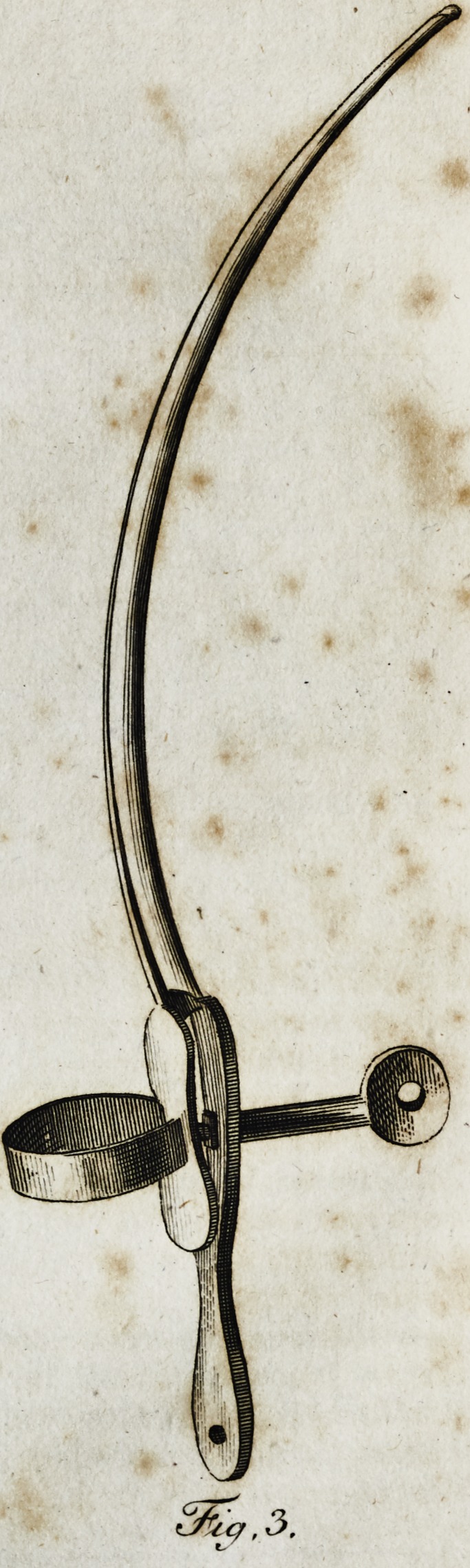# A Description of a New Instrument for Performing the Operation for the Fistula in Ano

**Published:** 1800-06

**Authors:** Thomas Whately

**Affiliations:** Member of the Royal College of Surgeons, in London


THE
Medical and Phyfical Journal.

VOL. III.] JUNE, 1800. [NO. XVI.

*To the Editors of the Medical and Phyfical Journal.*

GENTLEMEN,

IF the following paper be judged worthy of a place in your very ufeful Journal, it is at your fervice. I am,

GENTLEMEN,
Your very humble fervant,

*Bedford Row, May 9, 1800.* THO. WHATELY.

### *A Defcription of a new Inftrument for performing the Operation for the Fiftula in Ano.*

By THOMAS WHATELY, Member of the Royal College of Surgeons, in London.

SIMPLICITY of conftruction is certainly a great recommendation to all inftruments employed in furgical operations. We are not, however, to give the preference to any, merely on account of their fimplicity, as it may happen that the more complex may fometimes be better calculated to perform the operation in a proper manner; this is the cafe with Pott's inftruments for performing the operation for the radical cure of the hydrocele, which I know, from experience, anfwer better than the more fimple Seton lancet ufed by Mr. Hunter for the fame purpofe.

The ingenuity of artifts has been frequently exercifed in contriving an eligible inftrument for cutting for the fiftula in ano; an operation which confifts in dividing a portion of the rectum and of the adipofe membrane and the fphincter ani, fo as to lay open the finus or finufes which conftitute the difeafe. If thefe finufes are not completely laid open, or if the divifion be made in an improper place, the firft operation fometimes fails of making a perfect cure. Any of the inftruments which have been made for this purpofe, may anfwer the intention in particular cafes very well; but the blunt pointed crooked biftoury, recommended by that excellent furgeon Mr. Pott, certainly has the pre-eminence, and when the operation is performed after the recent burfting of abfceffes in thefe parts, or where the external orifice is open enough to admit readily the paffage of the knife, there is perhaps no inftrument more eligible.

There are, however, many cafes of fiftula in ano, in which I think a better inftrument may be ufed. It frequently happens in thofe of long ftanding, (which oftener come under the operator's care than the more recent ones) that the external orifice is very fmall; fometimes fcarcely large enough to receive the point of a common probe. In fome of thefe cafes, the fiftulous cavity leading to the gut may be eafily traced by a probe. In others, either on account of fmall windings in the cavity, or from other obftructions which the probe meets with in exploring it, a little time is required in the examination, in order to afcertain the direction and extent of the finus; and whether it communicates with the cavity of the rectum by a direct opening through the gut, or runs on its outfide only, without fuch a communication with its cavity. When the external orifice of the fiftula is very fmall, it will not be poffible in fome cafes, efpecially where a patient is timid, to pafs the probe-pointed knife fo as to meet the finger, without its wounding more or lefs fome of the parts in its paffage. If the true direction of the finus be not followed after the introduction of the knife, it muft be apparent to every one, that it cannot be explored without giving much unneceffary pain, by an inftrument that is liable to cut. And although it may not be difficult to pufh the inftrument within the cavity of the rectum, fo as to meet the operator's finger, yet it appears highly probable that this perforation may fometimes be made in a different part to that which was intended; an error which may occafion a failure in the cure. Sometimes we find the orifice of the fiftula fituated upon the buttocks, at the diftance of three or four inches from the anus: In this cafe, the external finus muft be in part opened by the knife, before the probe point of it can poffibly reach the operator's finger; and this may occafion fome difficulty in finding the true direction of the finus.

Thefe circumftances led me to adopt a new inftrument, which is the fubject of this paper. This inftrument confifts of a very narrow probe-pointed curved knife*, with a ring affixed

* This inftrument may likewife be made perfectly ftraight. In this form it will anfwer extremely well where the gut is to be flit not much above the fphincter;

Fig. 1.

Fig. 2.

Fig. 3.

affixed to its handle, (*fig.* 1,) and a ſheath on its blade, having a ſcrew fixed to one end of it, to confine the knife and the ſheath together, (*fig.* 2). By the handle of this ſcrew, the operator's aſſiſtant may draw off the ſheath from the knife. At the point of the ſheath there is a very fine diviſion in its central part, to the extent of a quarter of an inch, in order to prevent the edge of the knife being injured in withdrawing it. While both parts are fixed together, they make a perfectly ſmooth and uniform inſtrument, not unlike a curved probe. (*fig.* 3.) This inſtrument may be introduced into any fiſtulous orifice that will admit a common probe; and may be eaſily paſſed along the cavity with one hand, while its point is received by the fore finger of the other hand thruſt into the anus, in thoſe caſes where the gut is perforated by the diſeaſe. Where it is not perforated, this inſtrument, by means of the fore finger in its ring, can be very eaſily puſhed through it, as Mr. Pott juſtly obſerves may be done in a like ſituation with the probe-pointed knife. At this ſtage of the operation, the ſheath may be ſet at liberty in a moment by an aſſiſtant; after making a ſingle turn of the ſcrew, he may inſtantly, by its handle, withdraw the ſheath from the knife. The ſurgeon directly afterwards finiſhes the operation by dividing the gut, as with the probe-pointed knife.

With this inſtrument, (made within the laſt two months by Mr. Evans, in a very neat manner) I have performed the operation much to my ſatisfaction in five different ſubjects; the laſt of whom declares, that the whole operation gave him much leſs pain than the previous examination of the ſinus by the probe.

---

### *To the Editors of the Medical and Phyſical Journal.*

GENTLEMEN,

THE following Hiſtory of a Mal-formation is, I think, worthy of a place in your Journal. In the firſt place, it is no common mal-formation; it is, on the contrary, as Dr. Baillie remarks, a very uncommon one. (Morbid Anatomy, page 181).

---

ſphincter; but where this is to be done nearly to the extent of the fore finger, as will be neceſſary in ſome caſes, a ſtraight knife will, without great care, be apt to cut the operator's finger. It may alſo be made of any degree of curvature, or with a handle, of any ſhape or ſize, inſtead of the ring.

## Figures and Tables

**Fig.1. Fig.2. f1:**
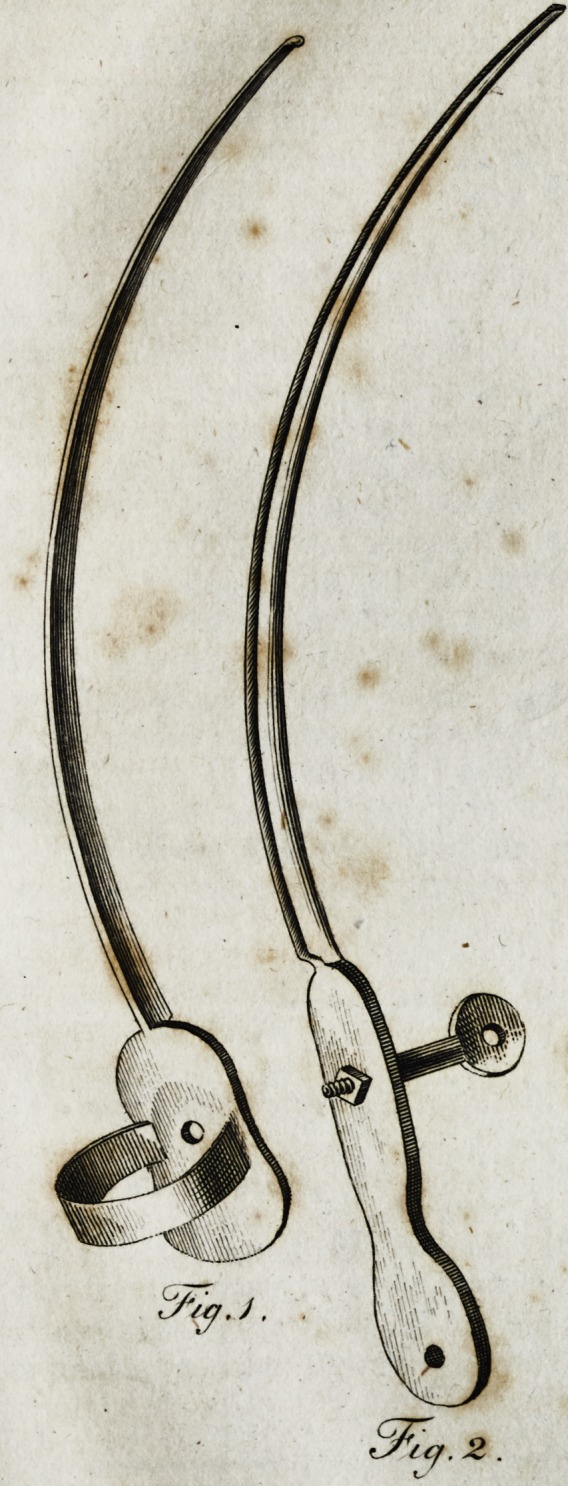


**Fig.3. f2:**